# Hypoxia-Induced CD36 Expression in Gastric Cancer Cells Promotes Peritoneal Metastasis via Fatty Acid Uptake

**DOI:** 10.1245/s10434-022-12465-5

**Published:** 2022-08-30

**Authors:** Tatsuya Aoki, Jun Kinoshita, Seiichi Munesue, Toshihide Hamabe-Horiike, Takahisa Yamaguchi, Yusuke Nakamura, Koichi Okamoto, Hideki Moriyama, Keishi Nakamura, Shinichi Harada, Yasuhiko Yamamoto, Noriyuki Inaki, Sachio Fushida

**Affiliations:** 1 Department of Gastrointestinal Surgery, Graduate School of Medical Science, Kanazawa University, Kanazawa, Ishikawa Japan; 2 Department of Biochemistry and Molecular Vascular Biology, Kanazawa University Graduate School of Medical Sciences, Kanazawa, Japan; 3 Center for Biomedical Research and Education, School of Medicine, Kanazawa University, Kanazawa, Japan

## Abstract

**Background:**

The lipid scavenger receptor cluster of differentiation 36 (CD36) has been shown to have a pro-metastatic function in several cancers. Adipose tissue, a favorable site for peritoneal metastasis (PM) from gastric cancer (GC), promotes this process by providing free fatty acids (FFAs); however, the role of CD36 in PM progression from GC remains to be elucidated.

**Materials and Methods:**

We evaluated CD36 expression in the GC cells under various conditions. CD36 overexpressing (CD36OE) MKN45 cells were prepared and their migration and invasive properties were assessed. A PM mouse model was used to investigate the biological effects of palmitic acid (PA) and CD36. Furthermore, we examined the clinical role of CD36 expression in 82 human PM samples by immunohistochemical staining.

**Results:**

Hypoxia markedly increased CD36 expression in GC cells. In normoxia, only CD36OE MKN45 cells treated with PA showed an increase in migration and invasion abilities. An increased expression of active Rac1 and Cdc42 was observed, which decreased following etomoxir treatment. Conversely, hypoxia increased those capacities of both vector and CD36OE MKN45 cells. In a mouse model transplanted with CD36OE MKN45 cells, more peritoneal tumors were observed in the high-fat diet group than those in the normal diet group. In clinical samples, 80% of PM lesions expressed CD36, consistent with hypoxic regions, indicating a significant association with prognosis.

**Conclusion:**

Our findings indicate that a hypoxia in the peritoneal cavity induces CD36 expression in GC cells, which contributes to PM through the uptake of FFAs.

Peritoneal metastasis (PM) is the most common cause of cancer recurrence and distant metastasis in gastric cancer (GC).^1^ The metastatic process occurs through a multistep cascade that is closely related to the molecular alterations present in both the cancer cells and the tumor microenvironment (TME).^2,3^ PM from GC preferentially occurs in adipocyte-rich organs such as the omentum, where adipocytes are the primary cellular components of the PM microenvironment. Emerging evidence indicates that adipocytes adjacent to invasive cancer cells, referred to as cancer-associated adipocytes, provide free fatty acids (FFAs), growth factors, and cytokines, and transdifferentiate into other stromal cells to alter tumor growth, metastasis, and drug responses.^4,5^ We also reported that adipocytes dedifferentiated into cancer-associated fibroblast-like immature cells upon contact with GC cells and increased the expression of IL-6 and plasminogen activator inhibitor-1, thereby enhancing the malignant potential of GC cells.^6^ However, molecular alterations in cancer cells induced by their contact with adipose tissue have not been completely elucidated.

The dominant energy-producing mechanism in cancer cells is glycolysis, which is known as the Warburg effect. However, tumor cells undergoing extracellular matrix detachment have decreased glucose uptake and lower glycolytic activity.^7^ Recently, fatty acid oxidation (FAO) was found to be the preferred alternative mechanism of energy production, which was dependent on mitochondrial oxidative phosphorylation in anoikis-resistant cells, such as circulating tumor cells and tumor-forming cells.^8,9^ In particular, the expression of cluster of differentiation 36 (CD36), a scavenger receptor that functions in the high-affinity uptake of long-chain fatty acids, was reported to be involved in the initiation of metastasis via FAO in oral squamous cell carcinoma.^10^ Accumulating evidence has confirmed that CD36 allows cells to take up lipids from the extracellular microenvironment and promotes FAO to produce ATP,^11,12^ potentially energizing tumor progression and metastasis.^13–16^

Resistance to anoikis is a hallmark of PM because intraperitoneal free cancer cells that have migrated from the primary tumor survive in an anchorage-independent manner.^17^ Therefore, GC cells require sufficient ATP generation, increased biosynthesis of biomolecules, and maintenance of an appropriate redox status despite the low oxygen (hypoxia) and nutrient levels within the peritoneal cavity. Little is known about the expression of CD36 and its mechanistic role in PM. Therefore, we hypothesized that CD36-dependent uptake and FAO using adipocyte-derived FFAs may be involved in the PM process.

In the present study, we used hypoxic conditions to assess the CD36 expression in GC cells and their contribution to the development of PM *in vitro* and *in vivo*. Furthermore, we also analyzed the CD36 expression in PM human samples using immunohistochemistry.

## Materials and Methods

### Ethical Approval

This study was approved by the Institutional Review Board of Kanazawa University Graduate School of Medical Science. Written informed consent was obtained from each patient. The present study was conducted in accordance with the ethical standards laid out by the institutional and national committees on human experimentation and the Helsinki Declaration of 1964 and later versions.

### Cells and Materials

GC cell lines MKN45, NUGC-4, and MKN74 were purchased from the Japanese Collection of Research Bioresources Cell Bank (Osaka, Japan). The cells were exposed to hypoxia (0.1% O_2_) using a BIONIX system (Sugiyama-gen Co.). Palmitic acid (PA; P9767, Sigma Aldrich, UK) was prepared as 2 mM stock solution by dissolving in 75% ethanol at 70 °C. Stock solutions were then added to 2% FA-free bovine serum albumin (BSA; 017-15146, FUJIFILM, Japan) to achieve the desired final FA concentration. PA or etomoxir (10 mM in dimethyl sulfoxide [DMSO], 124083-20-1, ChemScene, Japan) was added to the medium, and 75% ethanol and DMSO were used as respective controls.

### Immunofluorescence Staining

Immunofluorescence was performed as described in a previous study.^18^ Briefly, slides with the samples were incubated with CD36 antibody (NB400-144, rabbit, diluted 1:300, Novus Biologicals, Littleton, CO, USA) overnight at 4 °C. Immunoreactivity was visualized using anti-rabbit immunoglobulin (Ig) G secondary antibody conjugated with Alexa Fluor^®^ 546 (1:400; Molecular Probes/Invitrogen, Eugene, OR, USA). The cells were then incubated with DAPI for nuclear staining. The slides were observed using an immunofluorescence microscope (BX50/BS-FLA; Olympus, Tokyo, Japan).

### Flow Cytometry

Cells were incubated for 1 h with fluorescein isothiocyanate (FITC) anti-human CD36 antibody (336203; BioLegend, San Diego, CA, USA) at a 5:100 dilution in 100 μL of flow cytometry staining (FACS) buffer (2 mL fetal bovine serum (FBS)/100 mL phosphate-buffered saline [PBS]). Subsequently, the cells were washed twice with FACS buffer, followed by centrifugation at 1500 rpm for 3 min. Cell pellet was resuspended in 0.4 mL of FACS buffer and acquired on an Attune acoustic cytometer (Applied Biosystems, Life Technologies, Carlsbad, CA, USA). Analysis was performed using FlowJo v 9.6.1 software (FlowJo, Ashland, OR, USA).

### Cluster of Differentiation 36 (CD36) Plasmid Construction and Transfection

Complementary DNA (cDNA) was prepared and purified from messenger RNA (mRNA) extracted from human monocytes using a reverse transcription reaction (primers: 5′-GAGAATTCTTAACACTAATTCACCTCCT-3′ and 5′-GATCTAGATTATTTTATTGTTTTCGATC-3′) and amplified by polymerase chain reaction (PCR). After restriction digestion (EcoRI and XbaI; Takara Bio, Kusatsu, Shiga, Japan) of cDNA and vector (pCI-neo mammalian expression vector, E1841; Promega Corporation, Madison, WI, USA), the cDNA was ligated with the vector. cDNA expression vector was then transfected into MKN45 cells using Lipofectamine 3000 (Thermo Fisher Scientific, Waltham, MA, USA) according to the manufacturer’s instructions. Cells were cultured in the presence of G418 (400 μg/mL) post-transfection, and the G418-resistant colonies were picked after 2 weeks. Stably transfected cells were seeded in 96-well plates at a density of two cells/well for clonal expansion, and were gradually scaled up from 96 wells to 6 wells. Among the clonally expanded colonies, the groups with the highest expression of CD36 were selected. CD36 expression was verified using Western blotting and flow cytometry.

### Transwell Migration, Invasion Assays

Transwell migration and invasion assays were performed using 8 μm Transwell inserts (Migration Chamber: 140629, Lab Unlimited, Dublin, Ireland; Invasion Chamber: 354480, Corning Inc., Corning, NY, USA). Cells were suspended in serum-free RPMI-1640 medium without glucose or glutamine, containing 2% BSA and cultured in the upper chamber. 1% FBS-conditioned serum-free, no glucose, no glutamine RPMI-1640 with 2% BSA containing either 0.1 mM PA, 0.1 mM PA with 50 or 100 μM etomoxir, or respective controls, was added to the lower chamber. The cells that migrated or invaded the lower chamber of the inserts were photographed under a microscope (BX50/BS-FLA; Olympus), and five visual fields were randomly chosen to calculate the number of cells.

### Western Blotting

Western blotting was performed according to a previously reported standard protocol.^18^ The CD36 SR-B3 antibody (NB400-144, rabbit polyclonal IgG, diluted 1:1000, Novus Biologicals) was used. Rac1 and Cdc42 levels were measured using an Rac1/Cdc42 Activation Assay Kit (Sigma-Aldrich, Gillingham, Dorset, UK) according to the manufacturer's protocols. CD36- or vector-transfected MKN45 cells were incubated with etomoxir (100 μM) or 1% DMSO for 4 h, followed by exposure to PA (0.1 mM) or 3.75% ethanol in normoxia.

### Mouse Peritoneal Metastatic Model

All animal experiments were performed in accordance with Kanazawa University standard guidelines. Female immunocompromised BALB/c-nu/nu mice aged 4–6 weeks were maintained in a sterile environment. We randomly divided 20 mice into four groups and created GC peritoneal metastatic models using CD36 overexpressing (CD36OE) MKN45 cells or empty vector. Two groups of mice were fed a 60 kcal% high-fat diet (HFD; Research Diets, New Brunswick, NJ, USA) and the other two groups were fed a normal diet (ND). After 1 week of feeding, either 1 × 10^7^ vector or CD36OE MKN45 cells were intraperitoneally injected into mice in each group. All mice were sacrificed by cervical dislocation method 2 weeks after injection, and the size and number of peritoneal nodules. Plasma triglycerides were measured using the Mouse Triglyceride ELISA Kit (My BioSource, San Diego, CA, USA) according to the manufacturer’s protocols.

### Patient Population

Eighty-two patients who underwent biopsy or resection of PM of GC from 2000 to 2016 were selected as the study subjects. Of these, 67 were GC patients with PM at the time of initial presentation, and 15 were patients of peritoneal recurrence after R0 gastrectomy. Of the former, 44 patients underwent gastrectomy, 18 of whom had combined resections of organs such as small and large intestines due to the invasion of PM. In five of the peritoneal recurrence cases, bowel resection was performed for intestinal stenosis. Fifty-nine patients underwent biopsy or local excision of PM for diagnosis or macroscopic curative surgery, and 78 patients received chemotherapy, of whom 62 patients received systemic chemotherapy plus intraperitoneal chemotherapy. We examined the expression of CD36 and carbonic anhydrase IX (CAIX) in the primary and PM lesion samples of GC. We also evaluated 36 lymph node metastatic lesions in patients who underwent combined resection of PM and primary lesions. In addition, liver metastatic lesions were evaluated in 19 different patients.

### Immunohistochemical Staining

Immunohistochemical (IHC) staining was performed according to a previously reported standard protocol.^18^ CD36 antibody (NB400-144, rabbit, diluted 1:300; Novus Biologicals) and CAIX antibody (NB100-417, rabbit, diluted 1:500; Novus Biologicals) were used for the IHC assessment. Based on intensity, the staining was graded/scored as no staining (0), weak staining (1), moderate staining (2), and strong staining (3). The number of positively stained GC cells was divided into the following four ranges (proportion score): ≤5% (0), 6–25% (1), 26–50% (2), and >51% (3). The final staining score was calculated using the following formula: overall score = intensity score × proportion score. A final score of ≤4 was defined as negative staining and >4 as positive staining.

### Statistical Analysis

Statistical analyses were conducted using EZR statistical software version 1.32. We adopted the upper limit at our hospital as the cut-off value for C-reactive protein (CRP), carcinoembryonic antigen (CEA), carbohydrate antigen (CA) 19-9, and CA125. For prognostic nutritional index, platelet, and body mass index (BMI), a receiver operating characteristic curve was created based on the median survival time, and the nearest point in the upper left corner was set as the best value. The Chi-square test was used to determine the differences in CD36 expression and clinicopathological parameters, and the Kaplan–Meier method and log-rank test were used for survival analysis. Overall survival (OS) was calculated based on the date of diagnosis of PM or peritoneal recurrence. Univariate and multivariate analyses were performed using the Cox hazard model as a prognostic parameter. Statistical significance was set at *p* < 0.05.

## Results

### Hypoxia Increased CD36 Expression in Gastric Cancer (GC) Cells

**Fig. 1:**
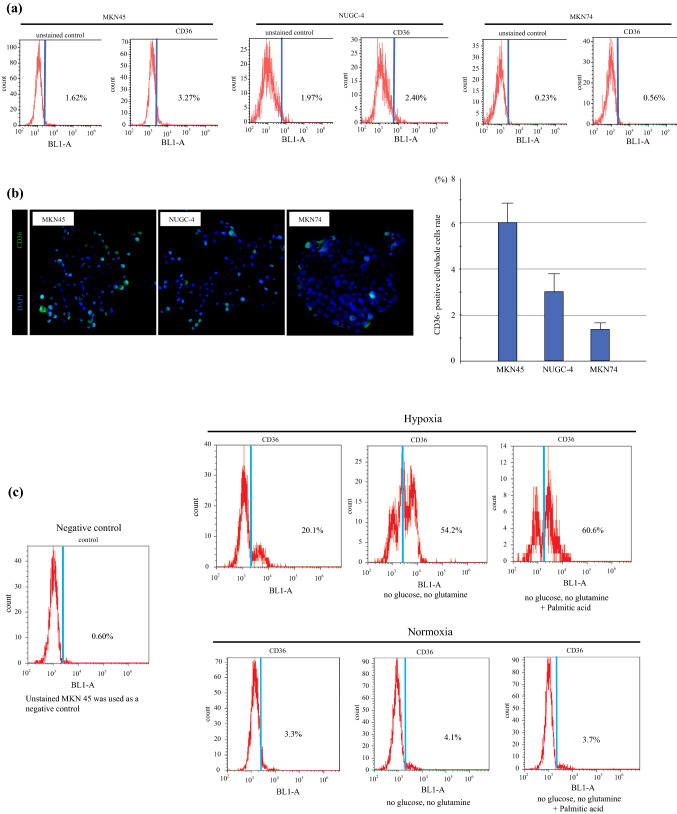
CD36 expression in gastric cancer cell lines. **a** Flow cytometry analysis showing the expression of CD36 in MKN45, NUGC-4, and MKN74 cells. **b** Immunofluorescence staining showing the expression of CD36 in MKN45, NUGC-4, and MKN74 cells. **c** Hypoxia increased CD36 expression in MKN45 cells. Flow cytometry analysis of CD36 expression in MKN45 cells after 24 h of incubation under conditions of hypoxia, no glucose, no glutamate, and palmitic acid administration

**Fig. 2:**
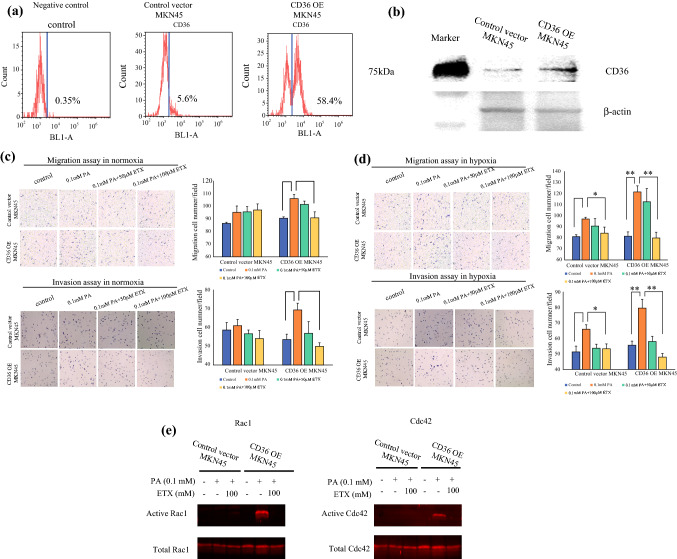
**a** Flow cytometric analysis of the transfection efficiencies of control vector or CD36 overexpression (CD36OE) MKN45 cells. **b** Western blot analysis of the transfection efficiencies of control vector or CD36OE MKN45 cells. **c** Transwell migration and invasion assay of vector and CD36 OE MKN45 cells after 0.1 mM PA/50 μM or 100 μM etomoxir treatment in normoxia. **d** Transwell migration and invasion assay of vector and CD36 OE MKN45 cells after 0.1 mM PA/50 μM or 100 μM etomoxir treatment in hypoxia. **e** Rac1 and Cdc42 were measured using an Rac1/Cdc42 Activation Assay Kit. Vector and CD36OE MKN45 cells were subjected to 16 h fatty acid-free starvation and were then treated with etomoxir (100 μM) or 1% DMSO for 4 h, followed by exposure with PA (0.1 mM) or 3.75% ethanol in normoxia. *PA* palmitic acid

Basal CD36 expression in MKN45, NUGC-4, and MKN74 cells was confirmed using flow cytometry and was found to be low, ranging from 0.5 to 3% (Fig. 1a). Similarly, fluorescence immunostaining revealed a fewer number of CD36-positive GC cells (Fig. 1b). Furthermore, CD36 expression was assessed in MKN45 cells under hypoxic conditions with no glucose, no glutamate media, and palmitate administration (Fig. 2). Under hypoxia, CD36 expression increased to 20.1% and further increased to 60.6% with no glucose, no glutamine, and the addition of PA. In contrast, no glucose, glutamine, or addition of PA increased the CD36 expression in MKN45 cells marginally under normoxia (Fig. 1c).

### Addition of Palmitic Acid Promoted GC Cell Migration and Invasion Through CD36 in Both Normoxia and Hypoxia

We aimed to assess whether exogenous PA promoted the migration and invasion of GC cells mediated by CD36. To mimic the heterogeneity and instability of CD36 expression in GC cells, we transfected the GC cell line MKN45 with CD36 expression plasmid and its control. Stably transfected cells showing similar CD36 expression levels, as analyzed by flow cytometry and Western blotting, were expanded from a large pool of samples and used for subsequent experiments (Figs. 2a, b).

We evaluated the migration and invasion properties of CD36OE MKN45 cells. Cells were divided into four groups according to the presence or absence of PA in the lower chamber and the presence or absence of etomoxir, a CPT1 inhibitor, in the upper chamber. Under normoxia, CD36OE cells treated with 0.1 mM PA showed increased migration and invasion capacity, which was inhibited by 100 µM etomoxir. Conversely, in the control vector cells, neither migration nor invasion capacity were altered by PA or etomoxir treatment (Fig. 2c).

Under hypoxia, the migration and invasive capacity of both the control vector as well as CD36OE MKN45 cells significantly increased with the addition of 0.1 mM PA. Furthermore, treatment with 50 and 100 μM etomoxir suppressed the enhanced migration and invasive ability observed with PA treatment in both the control vector and CD36OE MKN45 cells (Fig. 2d).

Rho GTPases, such as Rac1 and Cdc42, are well known for their roles in regulating cell migration and invasion. In CD36OE MKN45 cells, active Rac1 and Cdc42 were found to increase with PA stimulation, but were decreased post-etomoxir treatment (Fig. 2e). No changes were observed in the control vector cells under similar conditions (Fig. 2e). These findings suggested that the increase in active Rac1 and Cdc42 possibly mediates the enhanced migratory and invasive capacity in CD36OE cells compared with that in the control vector cells.

### High-Fat Diet Promoted the Growth of Peritoneally Implanted CD36 Overexpressing MKN45 Cells in Mice

**Fig. 3:**
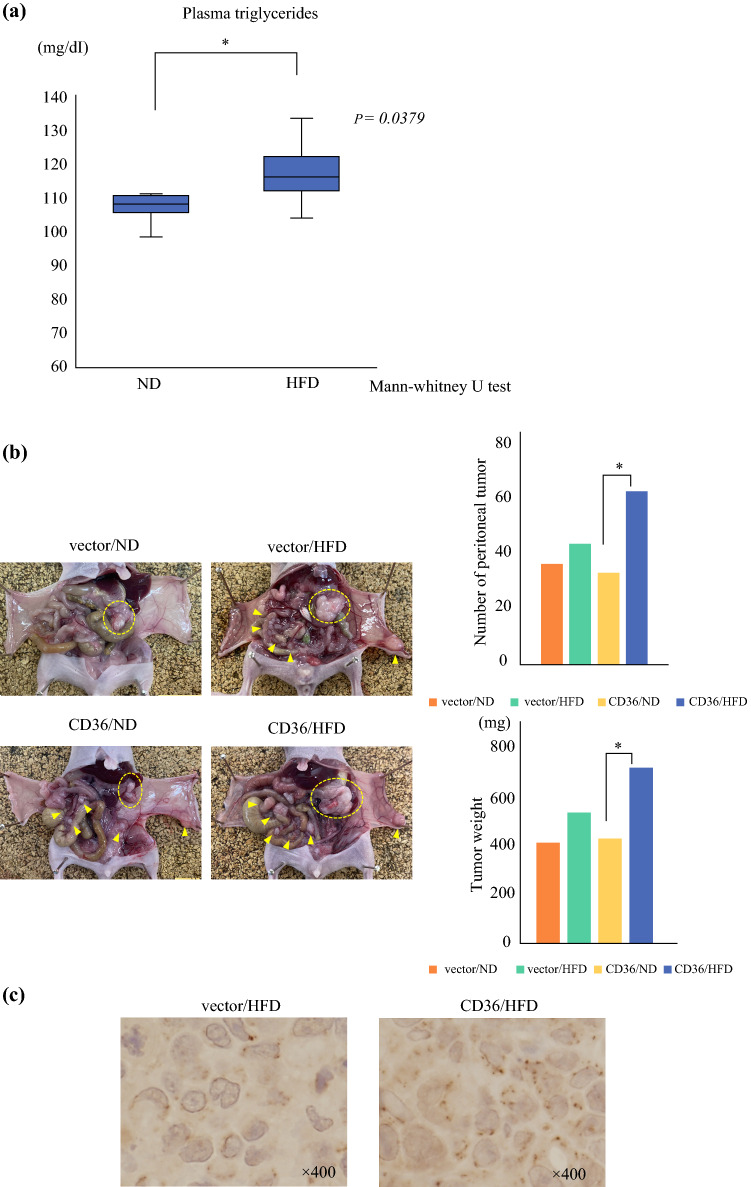
HFDs promoted the growth of peritoneally implanted CD36 overexpression (CD36OE) MKN45 cells in mice. **a** Plasma triglyceride levels in nude mice fed an ND or HFD. **b** Representative macroscopic images of peritoneal tumors (arrows and circles) in four groups of mouse models inoculated intraperitoneally with vector or CD36 OE MKN45 cells and fed an ND or HFD. The results of the number of peritoneal tumors and tumor weight are expressed as the mean ± SEM (*n* = 5, *p < 0.05). **c** Immunohistochemical staining of CD36 expression in peritoneal tumors of vector or CD36 OE MKN45 cells. *HFD* high-fat diet, *ND* normal diet, *SEM* standard error of the mean

To determine the differences in the dissemination ability induced by CD36-mediated FFA changes, control vector or CD36OE MKN45 cells were intraperitoneally injected into nude mice. An ND or HFD was then administered to the mouse model and their ability to form peritoneal tumors was compared. There were no significant differences in the body weights of mice in each group during the observation period (data not shown). The plasma triglyceride levels were higher in mice fed an HFD than those in mice fed an ND (Fig. 3a), indicating that FFAs promoted the growth of peritoneally implanted GC cells.

There were fewer peritoneal nodules in the vector cells/ND group compared with those in the vector cells/HFD group, however this difference was not significant (Fig. 3b). In mice with CD36OE MKN45 cell transplants, the HFD group had significantly more peritoneal tumor nodules and tumor weight than that in the ND group. Histological analysis of the xenografts also confirmed high CD36 expression in mice with CD36OE cell transplants (Fig. 3c).

### CD36 Expression in Clinical GC Tissue Samples

**Fig. 4:**
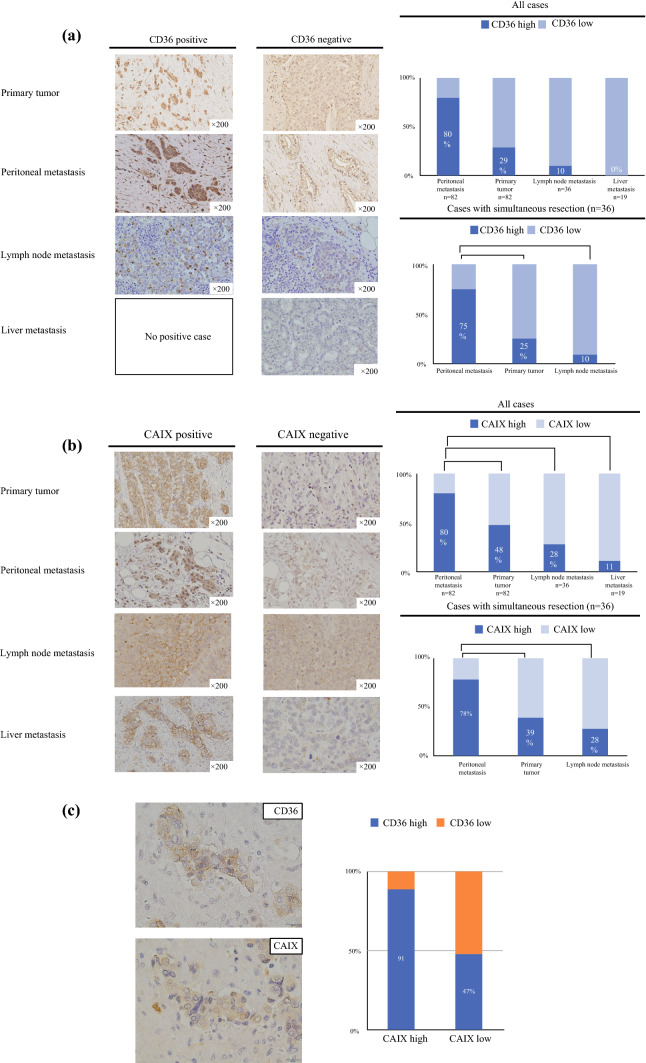
**a** Representative microscopic images of CD36 expression in primary tumors, and peritoneal, liver and lymph node metastases. Staining intensity of CD36 expression was calculated and the frequency of high expression in each organ was compared using the Chi-square test (*p < 0.05). **b** Representative microscopic images of CAIX expression in primary tumors, and peritoneal, liver and lymph node metastases. Staining intensity of CAIX expression was calculated and the frequency of high expression in each organ was compared using the Chi-square test (*p < 0.05). **c** Co-localization of CD36 and CAIX expression was evaluated in serial sections. CD36 expression is associated with CAIX expression (*p < 0.05)

CD36 expression increased in PM samples compared with their primary tumor counterparts (*p* < 0.05). In addition, CD36 expression in lymph node and liver metastatic tissues was significantly lower than that in PM tissue. The frequency of CD36 expression in the corresponding primary lesions, PM lesions, and lymph node lesions in the same cases was also comparable (Fig. 4a).

Since hypoxia modulates CD36 expression *in vitro*, we evaluated intratumoral hypoxia in primary tumors and metastatic tissues using CAIX staining as an endogenous surrogate marker of hypoxia. The CAIX staining index of peritoneal metastatic tissues was higher than that of the primary tumor, lymph node metastasis, and liver metastatic tissues. The expression of CAIX in the corresponding PM, primary tumor, and lymph node lesions in the same cases also showed similar results (Fig. 4b). Furthermore, we confirmed that CD36 expression was associated with CAIX expression using serial sections of the PM (Fig. 4c).

### High CD36 Expression and Overall Survival of GC Patients with Peritoneal Metastasis

We analyzed the relationship between CD36 expression and clinicopathological factors in GC patients with PM (Table 1), and found there was no significant difference in CD36 expression among histological differentiation in primary tumors and PM. Moreover, there was no difference in CD36 expression by tumor size between peritoneal specimens with combined bowel resection and those resected by biopsy or local excision. In addition, CD36 expression according to the location of resected PM lesions was examined, but there was no difference in CD36 expression between parietal and visceral peritoneal specimens.

**Table 1:** Relationships between CD36 expression and clinicopathological factors in patients with peritoneal metastasis

Factors		Number	CD36 expression		*p* value
			Low (*n* = 20)	High (*n* = 62)	
Age, years	>70	20	5	15	0.942
	<70	62	15	47	
Sex	Male	43	13	30	0.196
	Female	39	7	32	
Performance status	0	64	15	49	0.704
	1, 2	18	5	13	
Body mass index	<20.3	50	14	36	0.341
	>20.3	32	6	26	
Primary tumor differentiation	Intestinal	15	6	9	0.119
	Diffuse	67	14	53	
PM differentiation	Intestinal	11	5	6	0.080
	Diffuse	71	15	56	
First-onset case/recurrent case	First-onset case	67	14	53	0.119
	Recurrent case	15	6	9	
Size of PM	Bowel resection	23	3	20	0.135
	Biopsy or excision alone	59	17	42	
Location of resected PM	Visceral peritoneum	53	10	43	0.115
	Parietal peritoneum	29	10	19	
Gastrectomy	Yes	44	11	33	0.890
	No	38	9	29	
Chemotherapy	Yes	78	19	59	0.977
	No	4	1	3	
Intraperitoneal chemotherapy	Yes	62	15	47	0.942
	No	20	5	15	
P stage	P1a	12	4	8	0.435
	P1b, P1c	70	16	54	
CEA	>5	21	8	13	0.090
	<5	61	12	49	
CA19-9	>37	24	7	17	0.517
	<37	58	13	45	
CA125	>35	33	10	23	0.306
	<35	49	10	39	
Platelet	>223×10^3^	52	15	37	0.216
	<223×10^3^	30	5	25	
CRP	>1.0	24	6	18	0.934
	<1.0	58	14	44	
PNI	<40	48	12	36	0.879
	>40	34	8	26	

*PM* peritoneal metastasis, *CEA* carcinoembryonic antigen, *CA* carbohydrate antigen, *CRP* C-reactive protein, *PNI* prognostic nutritional index

**Fig. 5:**
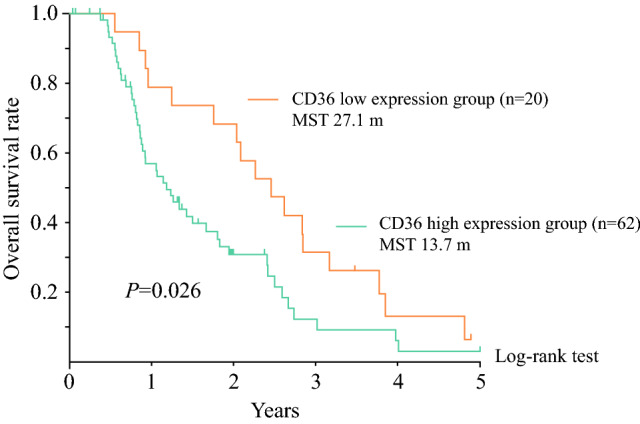
Kaplan–Meier analysis of overall survival based on CD36 expression in gastric cancer patients with peritoneal metastasis (*p* = 0.026)

As shown in Fig. 5, OS was significantly lower in PM patients with high CD36 expression than in those with low CD36 expression (*p* = 0.026).

To assess the prognostic potential of clinicopathological factors, including CD36, in GC patients with PM, univariate and multivariate analyses were performed using a Cox proportional hazards model (Table 2). In the univariate analysis, significant differences were found in chemotherapy, P stage (P1a/P1b, c), CA19-9, CRP, and CD36. Next, we performed multivariate analysis to determine the independent factors affecting OS. Variables with *p*-values <0.05 in the univariate analysis were included, and chemotherapy and CD36 were extracted as an independent prognostic factor for OS.

**Table 2:** Univariate and multivariate analysis for prognostic factors in patients with peritoneal metastasis

Variable		Number	Univariate analysis			Multivariate analysis		
			HR	95% CI	*p* value	HR	95% CI	*p* value
Age, years	>70 /<70	20/62	1.852	0.915–2.978	0.088			
Sex	Male/female	43/39	1.013	0.615–1.669	0.960			
Performance status	0/1, 2	64/18	1.578	0.853–2.919	0.146			
Body mass index	<16.85/>16.85	32/50	0.707	0.415–1.205	0.203			
Primary tumor differentiation	intestinal/diffuse	15/67	1.059	0.573–1.958	0.941			
PM differentiation	Intestinal/diffuse	11/71	1.375	0.618–3.058	0.435			
First-onset case/recurrent case	First-onset/recurrence	67/15	0.969	0.516–1.823	0.984			
Size of PM	Bowel resection/biopsy or excision alone	23/59	0.987	0.557 –1.750	0.987			
Location of resected PM	Visceral/Parietal	53/29	0.857	0.721–1.256	0.568			
Gastrectomy	Yes/no	44/38	0.7845	0.454–1.355	0.384			
Chemotherapy	Yes/no	78/4	0.113	0.0142–0.310	0.008	0.070	0.014–0.342	0.001
Ip chemotherapy	Yes/no	62/20	0.648	0.351–1.196	0.165			
P stage	P1b, P1c/P1a	70/12	2.091	1.020–4.287	0.044	1.569	0.715–3.461	0.264
CEA	>5/<5	21/61	1.354	0.736–2.489	0.328			
CA19-9	>37/<37	24/58	1.989	1.153–3.434	0.012	2.277	0.804–2.693	0.203
CA125	>35/<35	33/49	1.186	0.700–2.011	0.525			
CRP	>1.0/<1.0	24/58	1.834	1.023–3.287	0.039	1.782	0.998–3.397	0.056
PNI	<40/>40	48/34	1.613	0.939–2.769	0.080			
CD36 expression	High/low	62/20	1.863	1.064–3.262	0.029	2.678	1.118–3.983	0.021

*PM* peritoneal metastasis, *Ip* intraperitoneal, *CEA* carcinoembryonic antigen, *CA* carbohydrate antigen, *CRP* C-reactive protein, *PNI* prognostic nutritional index

## Discussion

The importance of CD36 in the regulation of proliferation, metastasis, and angiogenesis has been demonstrated, and its expression in primary tumors has been reported to be correlated with poor prognosis in different types of tumors.^19–21^ In this study, we report that a matched cohort of primary and PM tumors using human clinical samples showed upregulation of CD36 in PM tissue, and CD36 expression correlated with poor prognosis in patients with PM. We observed that CD36 expression was highly upregulated in hypoxia under glucose and glutamine deprivation and the addition of PA. In contrast, CD36 expression in response to these stimuli did not change under normoxia, suggesting that hypoxia is the primary driving force that induces CD36 expression in GC cells. As demonstrated by Kastelein et al. using three-dimensional whole-tumor imaging, hypoxic conditions in PM are due to poor perfusion of the microvasculature.^22^ Consistent with their findings, our clinical samples also showed more frequent and prominent CAIX-positive regions, reflecting hypoxia in PM relative to the primary tumor and lymphatic and liver metastases, and CD36 expression was upregulated, consistent with CAIX expression. Although tumors generally have an hypoxic environment in the central part as the tumor size increases, immunostaining of clinical specimens showed no difference in CD36 expression depending on the tumor size of the PM. We believe this is because most PM lesions, regardless of tumor size, have an hypoxic environment due to high intratumor pressure caused by abundant fibrotic stroma, which obstructs blood perfusion.^23^ In terms of the adipocyte environment, there was no difference in CD36 expression between the lesions on the adipocyte-rich visceral peritoneum, such as greater omentum or mesentery, and those on the adipocyte-poor parietal peritoneum. Comparisons based on BMI also showed no significant differences in CD36 expression. Thus, these results indicate hypoxia in TME was the most important factor affecting CD36 expression.

Under normoxia, CD36OE GC cells displayed enhanced migratory and invasive capacity and increased expression of active Rac1 and Cdc42 following PA treatment, which was suppressed by etomoxir. In hypoxia, similar changes were observed in control vector cells after PA treatment and etomoxir administration, although CD36OE cells showed a more prominent response. Our findings suggest that hypoxia in the peritoneal cavity enhances CD36 expression in GC cells and promotes the migratory and invasive abilities of cancer cells via the intracellular uptake of adipocyte-derived exogenous PA. Although HFD administration significantly increased peritoneal tumors in mouse models transplanted with CD36OE cells, the adipocyte environment, such as PM location and BMI, did not correlate with survival in human data. This discrepancy may be due to the extremely low visceral fat content of nude mice compared with humans. We suggest that in humans, FFA is supplied by adipocytes in the abdominal cavity, whereas in nude mice, FFA is supplied to CD36OE cells from systemic circulation as a result of an HFD.

Enhanced CD36 expression has been reported in hypoxic environments in non-malignant diseases. In hepatocytes, an hypoxic environment, as represented by obstructive sleep apnea, enhances CD36 expression on the plasma membrane and contributes to the onset of non-alcoholic fatty liver disease by increasing FFA uptake.^24^ In addition, hypoxia-inducible factor (HIF)-1α, which is considered the master regulator of oxygen homeostasis, upregulates CD36 expression and function in retinal epithelial cells and macrophages.^25,26^ Although hypoxia is considered a major stimulus for numerous malignancies through angiogenesis and tumor stemness, the molecular mechanisms governing the regulation of CD36 by hypoxia in malignant tumors are yet to be elucidated. Therefore, one of the most surprising findings of our study was that hypoxia induces CD36 expression in GC cells.

Although this study did not analyze the intracellular lipid metabolism of FFAs, several studies have reported increased lipid metabolism during hypoxia.^27,28^ Bensaad et al. reported that transcriptional regulation of fatty acid-binding proteins (FABP) 3 and FABP7 in glioma and breast cancer cells is induced by HIF-1α and leads to a significant lipid droplet (LD) accumulation during hypoxia.^29^ They demonstrated that triglycerides contained in LDs are degraded to generate FFAs when the cells need to produce ATP via FAO. FAO was recently found to be essential for the development of anoikis resistance and metastatic phenotypes in various cancers.^10,30–32^ Resistance to anoikis is thought to be a hallmark of PM as cells survive in a free state in the peritoneal cavity. Our study predicts that CD36-mediated FAO using FFAs derived from the peritoneal environment is involved in the development of PM.

## Conclusion

Our study demonstrates that (1) CD36 expression is upregulated in GC cells in response to hypoxia; (2) elevated CD36 expression contributes to the migratory and invasive abilities of GC cells and the peritoneal tumor growth using exogenous FFAs; and (3) PM exhibits high CD36 expression, which correlates with its prognosis. The regulation of CD36 expression by hypoxia may be a critical step in the development and progression of PM from GC. These data provide a valuable framework for future studies investigating the metabolic reprogramming of GC cells in the development of PM, where CD36 may be a promising new therapeutic target for PM.
